# Posterior urethral valves: Role of prenatal diagnosis and long-term management of bladder function; a single center point of view and review of literature

**DOI:** 10.3389/fped.2022.1057092

**Published:** 2023-01-06

**Authors:** Chiara Pellegrino, Maria Luisa Capitanucci, Valentina Forlini, Antonio Zaccara, Federica Lena, Maria Laura Sollini, Enrico Castelli, Giovanni Mosiello

**Affiliations:** ^1^ ERN EUROGEN Affiliated Center, Division of Neuro-Urology and Surgery for Continence, Bambino Gesù Childrens’ Hospital, IRCCS, Rome, Italy; ^2^ Pediatric Surgery Division, University of Genova, Genoa, Italy; ^3^ Division of Physical Rehabilitation, University of Tor Vergata, Rome, Italy; ^4^ Division of Neuro-Rehabilitation, Bambino Gesù Childrens’ Hospital, IRCCS, Rome, Italy

**Keywords:** posterior urethral valves, renal function, bladder outlet obstruction, bladder function, pediatric, urodynamic, prenatal diagnosis, eUrogen

## Abstract

Posterior Urethral Valves (PUV) are the most common cause of lower urinary tract obstruction. More severe forms are detected early in pregnancy (mainly type I), while other forms are usually discovered later in childhood when investigating lower urinary tract symptoms. Bladder dysfunction is common and is associated with urinary incontinence in about 55% (0%–72%). Despite the removal of the obstruction by urethral valve ablation, pathological changes of the urinary tract can occur with progressive bladder dysfunction, which can cause deterioration of the upper urinary tract as well. For this reason, all children with PUV require long-term follow-up, always until puberty, and in many cases life-long. Therefore, management of PUV is not only limited to obstruction relief, but prevention and treatment of bladder dysfunction, based on urodynamic observations, is paramount. During time, urodynamic patterns may change from detrusor overactivity to decreased compliance/small capacity bladder, to myogenic failure (valve bladder). In the past, an aggressive surgical approach was performed in all patients, and valve resection was considered an emergency procedure. With the development of fetal surgery, vesico-amniotic shunting has been performed as well. Due to improvements of prenatal ultrasound, the presence of PUV is usually already suspected during pregnancy, and subsequent treatment should be performed in high-volume centers, with a multidisciplinary, more conservative approach. This is considered to be more effective and safer. Primary valve ablation is performed after clinical stability and is no longer considered an emergency procedure after birth. During childhood, a multidisciplinary approach (pediatric urologist, nephrologist, urotherapist) is recommended as well in all patients, to improve toilet training, using an advanced urotherapy program with medical treatments and urodynamic evaluations. The aim of this paper is to present our single center experience over 30 years.

## 1. Introduction

Posterior urethral valves (PUV) are the most common cause of bladder outlet obstruction (BOO) in the pediatric population and are considered to be one of the life-threatening neonatal congenital pathologies. It leads to chronic kidney disease during childhood and adolescence in up to 32% of patients (1–3).

The incidence of PUV is currently 1 : 7.000–8.000 live births (4, 5). It occurs exclusively in males, with a variable degree of urethral obstruction. PUV are still classified according to the Young classification in type I (the most common), type II (not obstructive) and type III (6).

This congenital BOO at a critical time during organogenesis may produce a profound and lifelong effect on renal and bladder function. Severe fetal BOO can result in oligo/anhydramnios which may lead to pulmonary hypoplasia and Potter sequence. Based on the timing of presentation and the degree of BOO, the effects of PUV are variable in severity, ranging from a fatal condition in infancy to less severe forms presenting during childhood with lower urinary tract symptoms (LUTS). However, a later presentation can also be the result of a missed diagnosis (7, 8).

Prenatal diagnosis, improvement of neonatal respiratory assistance and a better understanding of bladder functional alterations has led to a significant decrease in mortality, from 50% to 5% (9).

Endoscopic PUV ablation is effective in resolving anatomical BOO, however sequelae on lower (LUT) and upper (UUT) urinary tracts may persist over the years and can deteriorate during childhood and adolescence (10–19). For this reason, the role of a multidisciplinary approach (including a neonatologist, pediatric urologist, nephrologist, and urotherapist) should be the standard of care in all patients, as well as careful nephro-urologic follow-up from the neonatal period up to adolescence. The aim of our paper is to present our current approach and how it has changed during time, in accordance with literature.

## 2. Prenatal diagnosis and management

Currently, up to 46.9% of PUV are discovered during routine prenatal ultrasound (US). When there is suspicion of PUV before the 24th week of gestation, it is associated with an increased risk of perinatal mortality and kidney disease (20, 21). In our hospital prenatal diagnostics of LUT malformations was started in 1986.

The main US findings suggestive for PUV are bilateral hydronephrosis, dilated bladder with a thickened wall (>3 mm) and a dilated posterior urethra (keyhole sign) (22). Furthermore, oligo/anhydramnios can also be present. The kidneys start to produce urine at the 10th week of gestation; by the second trimester, urine is the main source of amniotic fluid (AF). In case of fetal BOO with impaired renal function, reduction of urinary output can cause oligo/anhydramnios. Therefore, AF can be a possible prognostic factor of post-natal renal function (3, 23, 24). AF level is usually estimated by amniotic fluid index (AFI). An AFI between the 5th and 95th percentile is considered normal. Some authors believe that in case of suspected lower urinary tract obstruction (LUTO), caution should be exerted when an AFI is between the 5th–25th percentiles, with a possible association of post-natal renal failure (23). However, in our own experience this is not always the case, and a normal AFI is not always related to normal post-natal renal function (25). Additional US findings can be the presence of a perirenal urinoma, urinary ascites, bladder diverticula, or renal hyperechogenicity (sign of renal dysplasia) (26). Fetal urinary components, evaluated on samples obtained *via* vesicocentesis under US control, might be a further index of fetal renal function. Unfortunately, due to conflicting results, the risk of renal failure cannot be reliably predicted by fetal urine biomarkers (3, 27–29).

In our own experience and according to literature, the advantage of prenatal PUV diagnosis is mainly early identification of LUTO, since no scoring system is available at the moment to reliably predict postnatal renal function. In case of suspected PUV, the parents can be evaluated and counseled by a multidisciplinary fetal team, with the option of genetic counseling, and other associated anatomical anomalies can be assessed. In some cases, fetal magnetic resonance imaging is indicated to detect other congenital malformations or overcome difficulties of US evaluation due to maternal obesity or fetal position (30).

In selected patients and after thorough counseling of the parents, prenatal treatment may be proposed (31, 32). The most performed procedure is the placement of a vesico-amniotic shunt. This procedure was designed to decompress the urinary system, with the goal to reduce further kidney damage.

Despite the high expectations for vesico-amniotic shunting, a randomized international multicenter study (PLUTO-trial) showed inconclusive results on the real long-term benefit on renal function in patients who received this treatment. Moreover, the procedure had a complication rate of 21%–59%. Our experience showed similar results. Two other in-utero techniques have recently been proposed: valve ablation *via* cystoscopy and vesicostomy by open fetal surgery. However, both techniques are correlated with high maternal and perinatal morbidity (27, 32).

At the moment there are no clear parameters to predict future renal function. On the basis of prenatal suspicion of PUV, it is possible to plan the correct early, multidisciplinary treatment plan. In our opinion, treatment of patients with suspected PUV must be centralized in high volume pediatric urology centers. Fetal surgery can be proposed as an investigational therapy after extensive parental counseling in selected cases. A multicenter study is required in order to define the real advantages of these fetal procedures.

## 3. Post-natal management

**Figure 1:**
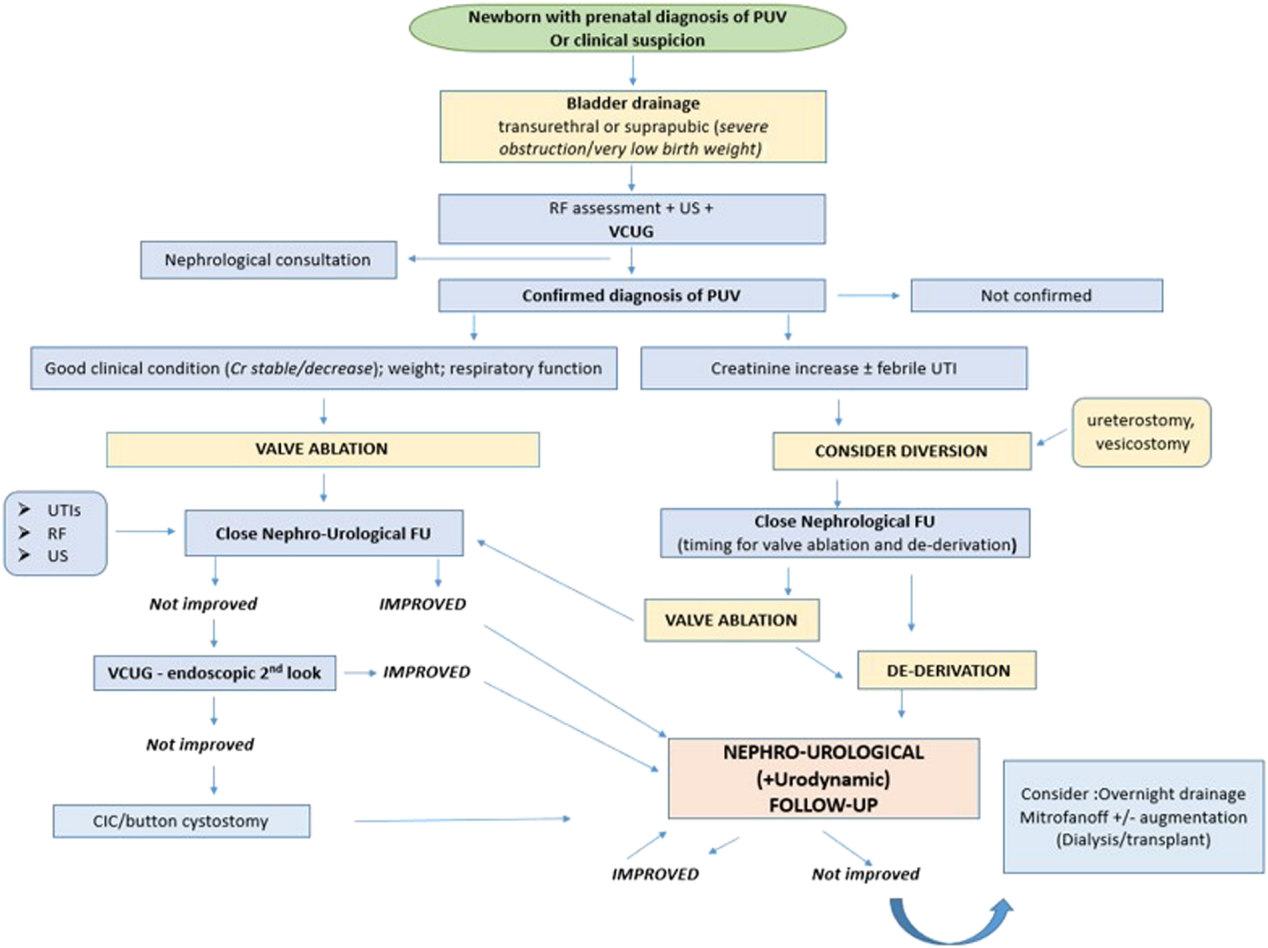
Post-natal management of PUV. PUV, posterior urethral valves; RF, renal function; US, ultrasound; VCUG, voiding cystourethrogram; UTI, urinary tract infection; FU, follow-up; Cr, creatinine.

Today, all newborns with prenatal suspicion of PUV are managed by a multidisciplinary team to define the urinary tract malformation, postnatal renal function, and supporting respiratory care when required. In a smaller percentage of cases, PUV is diagnosed at a later age, due to the presence of heterogeneous urinary symptoms, such as urinary tract infections, urinary incontinence, poor urinary stream, and enuresis. These patients may have a better renal outcome (33). However, Vasconcelos et al. did not find any significant differences in long-term outcome between patients with early and late PUV diagnosis. The only real difference between the two groups concerned urinary tract infections, which were more frequent in the post-natal diagnosis group. In contrast, Shields et al. published that patients with a late diagnosis of PUV have a worse prognosis, due to prolonged exposure of the kidney to higher pressures (7, 9). The conflicting results of these studies could be explained by the wide spectrum of PUV severity. It is plausible that patients with moderate to severe forms of BOO benefit from antenatal diagnosis because it can allow the planning of required nephro-urological management immediately after birth; otherwise, in patients with a minor form of BOO, the prognosis may not change. Therefore, it still remains unclear whether the presence of an antenatal diagnosis has a definitive correlation with the different long-term outcome parameters (Figure 1).

**Urological management:**

### 3.1. Bladder drainage

**Figure 2:**
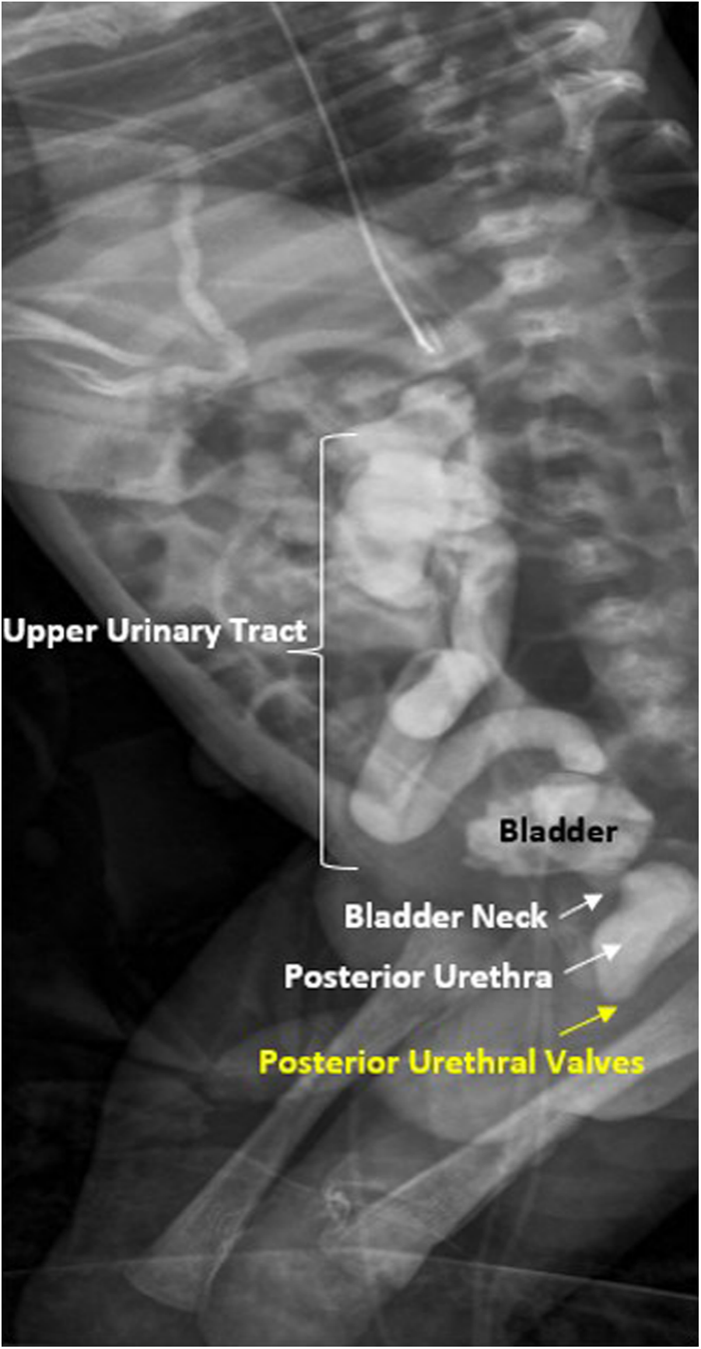
Voiding cistouretrography (VCUG) in newborn patient with posterior urethral valves with dilation of posterior urethra and vesico-ureteral reflux.

In a newborn with suspected diagnosis of PUV, the first urological goal at birth is to drain the bladder by placing a transurethral catheter, usually a small feeding tube (without a balloon). Subsequently, voiding cystourethrography (VCUG) must be performed to confirm the diagnosis of PUV (Figure 2) and to evaluate the correct position of the catheter inside the bladder (34). If transurethral placement is not possible, a suprapubic catheter should be placed.

### 3.2. Endoscopic ablation/incision

Once the patient has been stabilized (from a systemic, respiratory, and renal point of view), a cystoscopy can confirm VCUG findings and endoscopic ablation or resection of the valves should be performed (35). Neonatal cystoscopes make endoscopic treatment possible also in very small patients. Valve ablation in the first few months of life is associated with a better long-term outcome, in terms of urodynamics, vesicoureteral reflux (VUR), and improvement or resolution of hydroureteronephrosis (35–37).

Some newborns are poor candidates to undergo early ablation even with neonatal instruments because of the small caliber of their urethra. For these patients a progressive dilation technique of the urethra can be used, by placing a catheter of increasing caliber (36).

Valves are incised at 5 and 7 o'clock, and sometimes also at 12 o'clock. The techniques vary during time and are utilized according to the surgeon's preference: electrocautery incision, cold knife incision, or laser incision/fulguration. Few series have been published suggesting the holmium:YAG laser as a safe and effective alternative technique (38–40). In the past we utilized the holmium:Yag laser, but currently we use Thulium laser for valve incision, with good results in 25 patients. Until now, we experienced no postoperative hematuria and early voiding results are good. We are confident that this minimal invasive ablation is a preventive tool also in terms of late bladder dysfunction (41).

Complications of endoscopic treatment can occur with an incidence varying from 5 to 25% and can have significant morbidity, such as urinary extravasation, hematuria, and urethral stricture. Today, incision of PUV is recommended rather than ablation, to reduce postoperative strictures (38, 42–44).

After treatment of PUV we prefer to leave an indwelling catheter, and in severe forms with thick valves a suprapubic catheter as well.

The efficacy of endoscopic treatment should be highlighted through the finding of improvement in renal function, US, VCUG and/or endoscopic control (34). Some authors recommend early (and temporary) administration of oxybutynin after endoscopic valve ablation in infants to improve hydronephrosis and vesicoureteral reflux (45). However, we do not routinely use oxybutynin immediately after ablation in newborn and infants, but we administer it in older children and adolescents when indicated.

### 3.3. Bladder neck incision

The role of associated bladder neck incision (BNI) is controversial. Simultaneous valve ablation and BNI are proposed as an effective and simple approach to improve voiding dysfunction and short-term urodynamic outcomes, especially in PUV patients with a high bladder neck and bladder dysfunction (46). Very few studies are available to address these issues in long-term follow-up of children with PUV, and BNI has largely been abandoned due to the fear of incontinence and dry or retrograde ejaculation. Long-term impact of BNI on continence and ejaculatory function in adults who underwent concurrent PUV ablation and BNI during childhood has been assessed (47). In all these patients a single BNI at 6 o'clock was performed, not touching the verumontanum. The authors concluded that BNI was not associated with additional risk of incontinence and dry ejaculation in early adulthood and preserved antegrade ejaculation; these results have been confirmed in other series (48). Furthermore, ejaculatory and sexual dysfunction may be present in PUV patients regardless of BNI. These symptoms might be more attributable to the underlying congenital and structural anomalies (e.g., urethral anomalies, cryptorchidism, chronic renal failure, etc.) rather than the treatment approach (49, 50). In our hospital we have used BNI only in very few and selected cases, such as in PUV resection in older children or adolescents with a very hypertonic bladder neck, with a limited incision at 5 and 7 o'clock. In all cases we had improvement of voiding dysfunction without concerns in long-term follow-up.

However, we did not have a control group and therefore it is difficult to conclude whether the results are related to the resection of the PUV alone or to the additional BNI.

### 3.4. Urinary diversion

In some cases, the patient is too small or clinically unstable to perform valves ablation after birth. In these cases, urinary diversion should be achieved by placing a transurethral/suprapubic catheter or making a temporary vesicostomy. However, there is still an important debate today about the usefulness of a vesicostomy in patients with PUV. Some authors believe that constructing a surgical vesicostomy can alter bladder function and thus should be avoided, while others consider it safe (37, 51, 52).

Even when an effective resolution of anatomical BOO is achieved, some patients develop progressive decline in renal function and febrile urinary infections (UTIs). This can be associated with persistent upper urinary tract obstruction due to a uretero-vesical obstruction as a result of a thick bladder wall, or due to high-degree vesicoureteral reflux. In these cases, creation of a high urinary diversion (ureterocutaneostomy or pyelocutaneostomy) can be considered to improve urinary drainage and decompress the urinary system (34). In a small series good results were shown after performing an infraumbilical mini-vesicostomy to establish a long-term clean intermittent catheterization (CIC) and a nocturnal drainage of the bladder (53).

We prefer to use a percutaneous suprapubic vesicostomy and try to avoid surgical vesicostomy as much as possible. When severe VUR is present, ureterocutaneostomy is considered. For long-term management, button cystostomy is an option as well.

### 3.5. Vesico-ureteral reflux

The incidence of vesicoureteral reflux in PUV patients is high, varying from 48% to 66%. Although the incidence of VUR in PUV is relevant, spontaneous resolution can occur in 27%–79% of cases, from 2 weeks to more than 1 year after valve ablation (54–57). Several studies documented that VUR and recurrent UTI are not associated with worse renal outcomes (58, 59).

Deterioration of renal function in refluxing unit appears to be related to antenatal congenital abnormalities of the kidney in association with BOO (60). The EAU–ESPU guidelines suggest antibiotic prophylaxis in boys with high-grade VUR and PUV (34). Based on the literature, we usually have a conservative approach for VUR in PUV boys (antibiotic prophylaxis, oxybutynin) during the first 3 years of life. Circumcision can be effective as well in prevention of febrile UTI's (61). Surgery (such as vesicoureteral reimplantation or urinary diversion) is considered for the most severe cases (recurrent febrile UTI, progressive renal failure) (62–64). This is also supported by the fact that the complication rate of ureteral reimplantation in PUV patients is high (45%–67%) (59).

If surgical correction is required, we primarily perform endoscopic treatment of VUR or alternatively an extra-vesical reimplantation (open or laparoscopic) (65). Another alternative could be urinary diversion using a button cystostomy. We believe that in PUV patients with VUR, the use of videourodynamics during follow-up is very important.

## 4. Follow-up

It is well established that despite complete and timely resolution of anatomic BOO, long-term sequelae on upper and lower urinary tract function can occur (37), leading to renal failure and urinary incontinence. Studies on multivariate analysis of risk factors for long-term renal failure showed that nadir serum creatinine and bladder dysfunction are the main prognostic factors for renal function impairment in patients with PUV (10, 11, 66, 67). For these reasons a regular nephrological and urological follow-up is advised (68–70).

There are currently no standardized guidelines or best practice recommendations for evaluating long-term renal and bladder function in PUV patients. However, yearly evaluation of creatinine level, ultrasound (US) evaluation of urinary tract morphology, and lower urinary tract function exams using invasive and non-invasive urodynamic studies are recommended.

Sexual function and fertility in PUV boys seem comparable to the general population. Although there are only few reports in the literature, normal sexual function and semen analysis compatible with paternity was shown in most patients (50). The presence of possible risk factors, such as altered urethral anatomy and end-stage renal failure, must certainly be taken into consideration (48, 49).

### 4.1. Renal function, chronic (CKD) and end stage (ESKD) kidney disease and kidney replacement therapy (KRT)

Deterioration of renal function is one of the main concerns among children with PUV. In recent reports from two multicenter cohorts (71, 72) 7%, 12% and 20% of children needed KRT at the ages of 1, 5 and 15 years, respectively. In another study, after 10 years, 50% progressed to CKD stages 2–4, and 15% needed KRT (73). From 2002 to 2011, 11% of children with KRT in North America originally had PUV (74). Although the data are variable throughout different studies, the high percentage of patients with CKD (up to 50%) and ESKD (up to 20%) justify regular follow-up, as is performed in our hospital.

**Figure 3:**
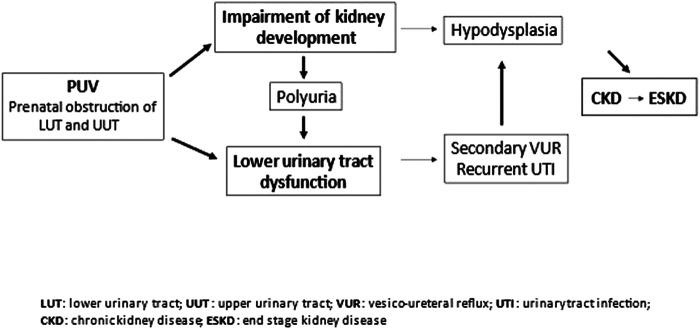
Pathophysiology of chronic kidney disease in posterior urethral valves. Posterior urethral valves (PUV) lead to obstructions of the urinary tract, with consequent impairment of kidney and bladder function development. Impairment in kidney development may result in hypodysplasia with a reduced nephron mass, and chronic kidney disease. Chronic kidney disease can progress to end-stage kidney disease. Lower urinary tract dysfunctionn can be aggravated by polyuria of the impaired kidneys and cause secondary vesicoureteral reflux and recurrent urinary tract infections. The latter may cause parenchymal scars in the hypodysplastic kidneys and accelerate renal failure progression.

Two main factors mainly contribute to chronic renal failure progression: impairment of prenatal nephrogenesis and bladder dysfunction (Figure 3).

In early infancy renal failure seems mainly caused by congenital renal dysplasia, due to back pressure from bladder outlet obstruction (3). According to this concept, the "pop-off" mechanism has been suggested to explain unilateral preserved renal function in presence of bladder diverticula, urinary extravasation with urinary ascites, perirenal urinoma and VURD Syndrome (VesicoUreteral Reflux Unilateral Renal Dysplasia) (71).

Measurements of plasma creatinine and creatinine clearance (75–79), urinary protein excretion and prevalence of hypertension (11, 80–82) are the most common markers for follow-up in PUV patients.

In long-term follow-up, reduced urine concentration capacity can be observed in about 75% of PUV children, with tubular damage that deteriorates with age (83–85), leading to polyuria (83, 86). The increased urinary volume in a dysfunctional bladder can cause further deterioration of the upper urinary tract. Overnight drainage in these patients should be suggested (83, 86, 87). In some of these patients the placement of a button cystotomy can be useful, since the urethral catheterization is not always easy to perform or accepted (due to a high bladder neck and a sensitive urethra) (88).

**Figure 4:**
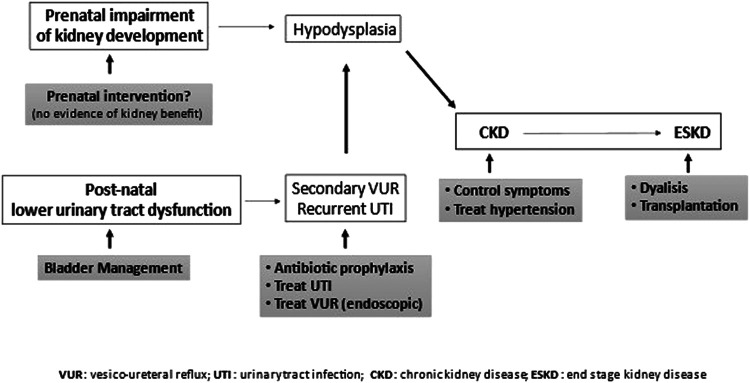
General principles of CKD and bladder dysfunction treatment. Prenatal intervention (vesicoamniotic shunting, valve ablation *via* fetal cystoscopy, vesicostomy by open fetal surgery) yields no renal benefit. Since lower urinary tract dysfunction increases risks of developing chronic kidney disease, it should be treated. Urinary tract infections should be prevented (prophylaxis) and treated. Symptomatic secondary vesico-ureteral reflux, which persisted despite management of lower urinary tract dysfunction, should be treated preferably with endoscopic technique. CKD progression should be slowed down, treating symptoms and hypertension, n end-stage kidney disease, dialysis or preferably transplantation is necessary.

When renal function is impaired, general principles of CKD treatment should be applied (89) in addition to the urological management of bladder dysfunction (Figure 4) (90).

When renal transplantation is required, the presence of a dysfunctional bladder may affect graft survival and function. Ureteral implantation can be more challenging, due to the increased bladder thickness, with subsequent risk of ureteral stenosis and obstruction of the upper urinary tract. According to other studies, however, initial treatment of the valves does not affect bladder dysfunction nor the survival of the graft (91–93).

The risk of urinary tract infection after kidney transplantation is reported higher in patients who underwent bladder augmentation. For this reason, many authors suggest to perform this surgical procedure only in case of an unresponsive low compliance bladder.

Bladder augmentation can be performed after kidney transplantation, with a similar complication rate when compared to preemptive augmentation (94, 95). On the other hand, the general consensus is that graft survival is better when management of bladder dysfunction is optimized, for instance by the execution of the CIC (transurethral or *via* a catheterizable channel) (96).

### 4.2. Lower urinary tract dysfunction (LUTD)

In the 1980s the concept of Valve Bladder Syndrome was introduced. It was described as a thick-walled, poorly compliant bladder with high resting pressure, resulting in hydroureteronephrosis and renal failure (97). Several studies investigating the urodynamics of these bladders identified three main different urodynamic patterns of valve bladder dysfunction: detrusor overactivity/hypercontractility, normal/low compliant bladder, and detrusor hypocontractility, leading to myogenic failure due to overdistention. These three urodynamic patterns are an evolution of the same LUTD, changing in relation to the age of the patient, and sometimes overlap (15–19).

Infants with valve bladders have reduced functional bladder capacity, and detrusor overactivity. During the following 1–3 years, bladder capacity increases, overactivity persists, and bladder emptying is incomplete. At 4–12 years, there is a further increase in bladder capacity; in these ages, overactivity disappears, and detrusor contractility decreases during voiding with incomplete bladder emptying (98). In PUV patients with myogenic failure, an elevated posterior bladder neck has been described, supporting the need of bladder neck incision (99). At puberty, the development of the prostate gland can also contribute to a deterioration of bladder emptying.

Based on our studies, urodynamic follow-up has a central role in PUV patients. We analyzed the urodynamic assessments of 48 patients with PUV, finding anomalies in 71% (16). In patients younger than 5 years of age, invasive urodynamic studies (cystometry or videourodynamics in case of associated VUR) are necessary to evaluate LUTD. Videourodynamics can provide the best details about the function of detrusor and bladder neck, detrusor-sphincter coordination, and urethral patency. This invasive evaluation must be considered if non-invasive urodynamic study suggests voiding dysfunction, to address the best bladder dysfunction management, according to bladder filling and emptying records. Urethral catheterization for the urodynamic study is regularly not accepted in PUV boys. For this reason, we prefer to perform this evaluation with two suprapubic 5 Ch catheters (Cistofix® B. Braun), positioned under sedation with cystoscopic control.

If valves remnants are present, laser ablation is performed during endoscopy (100). In our follow-up, standard urodynamic evaluations are scheduled at 1, 3 and 5 years. In older toilet-trained patients, non-invasive urodynamic evaluation is primarily performed (101), allowing greater adherence to urodynamic follow-up (102, 103). Invasive urodynamics is reserved for patients with symptoms and signs of progressive deterioration of LUTD or renal function.

### 4.3. Management of lower urinary tract dysfunction

In our opinion, treatment of valve bladder dysfunction should be early and aggressive. It includes behavioral modifications and timed voiding, medical therapy (anticholinergics or alpha-blockers), bladder neck incision, clean intermittent catheterization and overnight catheter drainage, or more invasive approaches such as botulinum toxin injections, creation of a catheterizable channel and bladder augmentation.

When anticholinergics were found to be ineffective to treat overactivity, we use intradetrusor injection of *Onabotulinum Toxin A* (Botox®), with good results (104). However, there was no improvement in the outcome of these patients from treatment with Botox® injections in the bladder neck (105). *Alpha-blockers* can be used to reinforce timed voiding (106). *Biofeedback therapy* and home *pelvic floor exercises* could provide significant and durable results for persistent LUTD after valve ablation in patients with side effects or poor response to drugs, with an overall consistent good response in 70% of the patients (107).

*Clean intermittent catheterization (CIC*) must be considered in some cases for emptying the bladder (108, 109). The sensitive perineum and the dilated posterior urethra can make CIC difficult in a child, leading to non-compliance and deterioration of the upper urinary tract. An abdominal *catheterizing channel* may be a viable alternative to keep the bladder empty (110). Another catheterization option quite recently proposed was the “button cystostomy” (88). In our experience this option can be very useful and well accepted by patients and families especially when *nocturnal bladder drainage* is required (111, 112).

*Bladder augmentation* may be required in PUV patients with small and low-compliant bladder, when medical management fails to prevent deterioration of renal function or continence (113, 114). Metabolic consequences and long-term complications should be taken into consideration for pediatric patients (115–117). In the long-term follow-up of pediatric augmentations, the incidence of malignancy in the augmented bladder should not be neglected (118). As reported by Hussmann et al., 12% of PUV patients (2/18 patients) who underwent bladder augmentation and kidney transplantation developed an invasive poorly differentiated adenocarcinoma of the augmented portion of the bladder. The tumors developed after a long time (30 years). It is not known whether this was a consequence of primary disease, of bladder enlargement or of immunosuppressive therapy. Repeated endoscopic control in adult patients is now routinely performed. We prefer to start endoscopic control and bladder wall biopsies early in childhood.

## Discussion and conclusion

Management of children with PUV is a continuous process starting with antenatal detection, followed by valve ablation, then subsequent control and management of bladder and renal function. Data on urinary markers beyond microalbuminuria and β2-microglobulin are scarce, and novel postnatal biomarkers such as proteomic data are missing and should further be investigated. Prenatal intervention yields no benefit on renal function. The main advantage of prenatal diagnosis is the opportunity to centralize patients with PUV and using a multidisciplinary approach already before birth. The best postnatal predictor for progression to CKD is the nadir serum creatinine. Even after a successful valve ablation, lower urinary tract dysfunction occurs in a significant percentage of patients, which can lead to further decrease of renal function. Urodynamic studies allow early identification of the specific type of bladder dysfunction, therefore enabling the correct planning of the right treatment. Furthermore, urodynamics can help to recognize subsequent changes in bladder function during follow-up, thus allowing management to be changed when necessary. When conservative therapy fails to solve impaired bladder emptying, intermittent catheterization should be started either transurethral or through a catheterizable channel. Bladder augmentation should be reserved to a selected group of patients. Whether bladder augmentation must be performed before or after renal transplantation remains a topic of discussion.

Valve ablation is not an emergency procedure, therefore PUV patients must be referred to highly specialized centers which are able to offer a multidisciplinary approach, such as the centers identified in ERN Eurogen (119).

This work is generated within the European Reference Network for Rare Urogenital Diseases and Complex Conditions (ERN EUROGEN).
